# Underweight Among Children Aged 6–59 Months in Transformed and Nontransformed Districts of East Gojjam, Ethiopia: A Community‐Based Comparative Cross‐Sectional Study

**DOI:** 10.1155/jnme/9041634

**Published:** 2026-09-22

**Authors:** Berhanu Abebaw Mekonnen, Kassahun Bishaw Anagaw, Netsanet Fentahun

**Affiliations:** ^1^ Department of Nutrition and Dietetics, School of Public Health, College of Medicine and Health Sciences, Bahir Dar University, Bahir Dar, Ethiopia, bdu.edu.et

**Keywords:** district transformation, Ethiopia, health extension program, under-five children, underweight

## Abstract

**Background:**

Underweight, a composite indicator of both acute and chronic undernutrition, remains a major public health concern among under‐five children in Ethiopia, contributing to increased morbidity, mortality, and impaired growth and development. Ethiopia’s Health Extension Program promotes transformed districts to improve child health and nutrition outcomes; however, evidence comparing underweight between transformed and nontransformed districts remains limited. This study assessed the prevalence of underweight and its associated factors among children aged 6–59 months in East Gojjam Zone, Ethiopia.

**Methods:**

A community‐based comparative cross‐sectional study was conducted among 627 children selected from transformed and nontransformed districts. Data were collected using structured questionnaires covering sociodemographic characteristics, household environment, childcare practices, and food security. Anthropometric measurements were obtained following World Health Organization standards, and underweight was defined as weight‐for‐age Z‐score < −2 standard deviations. Bivariable and multivariable logistic regression analyses were performed to identify independent predictors of underweight, with statistical significance set at *p* < 0.05.

**Results:**

The overall prevalence of underweight was 22.7% (95% CI: 19.0–26.0). Although underweight was more common among children in nontransformed districts (27.7%) compared with transformed districts (17.8%), this difference was not statistically significant. District transformation status was not independently associated with underweight after adjustment. Factors significantly associated with underweight included recent diarrheal illness (AOR = 2.49; 95% CI: 1.56–3.98), household food insecurity (AOR = 2.67; 95% CI: 1.60–4.46), low household income (AOR = 3.77; 95% CI: 2.34–6.08), inequitable intrahousehold food allocation disadvantaging children (AOR = 1.64; 95% CI: 1.02–2.65), and use of unprotected drinking water sources (AOR = 1.82; 95% CI: 1.10–2.99).

**Conclusions:**

Underweight remains a substantial public health problem in the study area and is driven primarily by household socioeconomic conditions, food insecurity, unsafe water access, and childhood illness rather than district transformation status alone. Strengthening multisectoral interventions that address poverty, food security, equitable child feeding, water safety, and infection prevention is essential to reduce underweight and improve child health outcomes.

## 1. Introduction

Underweight is defined as a child’s weight‐for‐age being substantially below that of the World Health Organization (WHO) reference population and is a key indicator of childhood undernutrition, reflecting acute, chronic, or combined forms of malnutrition [1, 2]. Children who are severely underweight face higher risks of morbidity, mortality, and impaired cognitive development [3, 4]. The most vulnerable age group is children aged 6–59 months, a critical period for growth and development, during which inadequate nutrition can lead to stunting, wasting, and underweight [5, 6].

According to the 2025 Joint Child Malnutrition Estimates by UNICEF, WHO, and the World Bank, child undernutrition remains a major global public health challenge. Globally, an estimated 150.2 million children under 5 years of age were affected by stunting, while 42.8 million children experienced wasting in 2024. Lower‐middle‐income countries bear a disproportionate share of this burden, accounting for nearly two‐thirds of all stunted children and about three‐quarters of those affected by wasting. Underweight, as a composite indicator that reflects both chronic and acute forms of undernutrition, continues to serve as an important marker of child nutritional status, particularly in low‐ and middle‐income countries [7].

Globally, the prevalence of underweight has declined from 105.7 million in 2010 to 85.4 million in 2020; however, it remains a major public health problem, particularly in low‐ and middle‐income countries, contributing to approximately 45% of under‐five deaths [8–10]. In Ethiopia, the 2019 mini Demographic and Health Survey (EMDHS) reported that 21% of under‐five children were underweight, with a higher prevalence of 26.7% in the Amhara Region, indicating underweight among children is currently a major public health issue in the country [11, 12]. According to the WHO classification of public health significance, an underweight prevalence of 20%–29% among children under five years of age is considered a high public health problem, while a prevalence of ≥ 30% is considered very high [13]. Underweight compromises immunity, increases susceptibility to infections, and adversely affects cognitive development [14].

Multiple factors contribute to underweight, including inadequate dietary intake, childhood illnesses, low parental education, poor water, sanitation, and hygiene (WASH) conditions, suboptimal feeding practices, limited antenatal care (ANC), residence, and socioeconomic and cultural factors [15–17]. Childhood immunization improves child survival by preventing vaccine‐preventable infectious diseases and may indirectly contribute to improved nutritional status by reducing infection‐related disruptions in nutrient utilization and growth. Given the established relationship between infectious morbidity and childhood undernutrition, greater vaccination coverage may be associated with reduced underweight and improved growth outcomes in low‐ and middle‐income settings [18–20]. Household‐level determinants, such as food insecurity, unequal food distribution, low dietary diversity, and improper food handling, further exacerbate undernutrition among children [21, 22].

In response, Ethiopia’s Ministry of Health (MOH) has implemented the Health Sector Transformation Plan (HSTP), with District (Woreda) Transformation as a core agenda to improve primary healthcare, maternal and child health, sanitation, and nutrition outcomes. A district is classified as “transformed” when ≥ 85% of health and nutrition indicators, such as sanitation coverage, skilled delivery, community‐based health insurance (CBHI), and child nutrition programs, are achieved; nontransformed districts fall below this threshold [23–25].

Despite these interventions, evidence on the effectiveness of district transformation in improving child nutritional outcomes remains limited. Although previous studies, including those conducted in similar settings, have examined underweight and its associated factors among under‐five children, evidence comparing transformed and nontransformed districts while comprehensively assessing key programmatic and household‐level determinants, such as CBHI enrollment, growth monitoring participation, household food security, and dietary diversity, remains scarce. Therefore, this study aimed to compare the prevalence of underweight and its associated factors among children aged 6–59 months in transformed and nontransformed districts of East Gojjam Zone, providing evidence to inform targeted nutrition interventions and district‐level policy implementation.

## 2. Methods and Materials

### 2.1. Study Setting and Period

The study was conducted in East Gojjam Zone, Amhara Region, Ethiopia, including Shebel Berenta (transformed district) and Enarji Enawga and Goncha Siso Enesie (nontransformed districts). Data were collected from May 18 to June 18, 2022. East Gojjam Zone has 19 districts, of which three are transformed and 16 are nontransformed. The zonal town, Debremarkos, is located 300 km from Addis Ababa and 250 km from Bahir Dar, the capital of Amhara Regional State, Ethiopia.

### 2.2. Study Design and Population

A community‐based comparative cross‐sectional study was conducted. The source population included all children aged 6–59 months living in the selected districts. The study population consisted of mothers and their children aged 6–59 months residing in randomly selected kebeles of the districts. The study unit was an individual child paired with their mother within the household.

### 2.3. Eligibility Criteria

Households with at least one child aged 6–59 months and residing in the study districts for at least six months were included. Mothers who were seriously ill or unable to respond, and children with chronic illnesses other than malnutrition, were excluded.

### 2.4. Sample Size Determination

The sample size was determined using a two‐population proportion formula, assuming a 95% confidence level, 80% power, a design effect of 1.5, and a 10% nonresponse rate. Based on a previous study conducted in a similar setting, the prevalence of underweight among children aged 6–59 months was assumed to be 15.3% in transformed districts and 24.3% in nontransformed districts [17]. Assuming a one‐to‐one allocation ratio, the final sample size, after accounting for the design effect and anticipated nonresponse, was 627 children (313 from the transformed district and 314 from the nontransformed districts).

### 2.5. Sampling Technique and Procedure

A stratified random sampling technique was employed. East Gojjam Zone was stratified into transformed and nontransformed districts based on performance records obtained from the zonal health department. From a total of three transformed and sixteen nontransformed districts, one transformed district (Shebel Berenta) and two nontransformed districts (Enarji Enawga and Goncha Siso Enesie) were randomly selected. Within the selected districts, six kebeles were randomly chosen from each stratum. The total sample was proportionally allocated to the selected kebeles based on the number of children aged 6–59 months. Finally, individual children were selected from each kebele using simple random sampling from the community health information system (CHIS) lists.

### 2.6. Study Variables

The dependent variable was underweight, defined as a child’s weight‐for‐age Z‐score (WAZ) below −2 standard deviations as per the WHO reference population median [1]. Independent variables included sociodemographic characteristics such as child age and sex, family size, number of children, parental education, occupation, household income, marital status, religion, place of residence, and CBHI status. Environmental factors comprised household drinking water source, availability and use of latrines, and waste disposal practices. Childcare and feeding practices included duration of exclusive breastfeeding (EBF), complementary feeding, dietary diversity, intrahousehold food distribution, participation in growth monitoring, immunization status, deworming history, and recent childhood illness. Maternal health service–related variables included maternal age, ANC attendance, place of delivery, birth order, birth interval, postnatal care (PNC) utilization, maternal extra food intake during pregnancy and lactation, counseling received on child nutrition, and household food insecurity.

### 2.7. Operational Definitions

*A transformed district (Woreda)* was defined as a district that achieved at least 85% coverage of key health and sanitation activities, including improved sanitation, skilled delivery services, CBHI enrollment, maternal and child health improvements, and model school implementation; districts not meeting this threshold were classified as nontransformed [23]. *An improved latrine* refers to any sanitation facility that hygienically separates human excreta from human, animal, and insect contact and includes a handwashing facility with soap [26]. *A protected water source* was defined as a drinking water source that is likely protected from external contamination, particularly fecal matter [26]. Diarrhea was defined as the passage of three or more loose or watery stools within a 24 h period, as reported by the mother [17].

### 2.8. Data Collection Instruments and Procedures

Data were collected by a trained team of eight diploma nurses and supervised by two BSc public health officers selected based on their familiarity with the study area, proficiency in the local language, and interest in the study. A semistructured questionnaire, adapted from the EMDHS, the WHO Infant and Young Child Feeding (IYCF) guideline, the Household Food Insecurity Access Scale (HFIAS), and previous community‐based studies conducted in Ethiopia, was used to collect information on sociodemographic characteristics, household environmental conditions, child care and feeding practices, maternal health service utilization, and household food security [5, 11, 17, 27, 28]. The questionnaire was translated into Amharic and pretested before data collection to ensure clarity, consistency, and cultural appropriateness.

Anthropometric measurements were conducted following WHO (2006) standards. Children’s ages were obtained from birth certificates, vaccination cards, or a local‐events calendar. Weight was measured using a digital scale to the nearest 0.1 kg, with minimal clothing and no shoes. For children unable to stand, weight was calculated by subtracting the mother’s weight from the combined mother–child weight.

Dietary diversity was assessed using a 24 h recall following the WHO IYCF guideline. Food intake was grouped into seven categories: starchy staples; legumes and nuts; vitamin A‐rich fruits and vegetables; other fruits and vegetables; eggs; dairy products; and flesh foods. Children consuming four or more categories were classified as having adequate dietary diversity [27]. Household food security was assessed using the HFIAS [28].

### 2.9. Data Quality Control Measures

The questionnaire was prepared in English, translated into Amharic, and back‐translated to English by bilingual experts to ensure consistency. All data collectors and supervisors received 2 days of training on study objectives, data collection procedures, anthropometric measurement techniques, and ethical considerations. A pretest involving 5% of the sample was conducted in Enemay District from April 28 to 30, 2022, to assess clarity, consistency, and validity of the tool, and revisions were made based on feedback. Anthropometric standardization was conducted on 10 children following the SMART standardization procedure [29]. Questionnaires were checked daily for completeness and consistency. Weight scales were calibrated to zero before each measurement, and reliability was routinely monitored. Data entry, coding, and cleaning were performed by the principal investigator, with inconsistencies cross‐checked against the original questionnaires to ensure accuracy.

### 2.10. Data Processing and Analysis

Data were entered into EpiData Version 3.1 and exported to SPSS Version 25 for analysis. Anthropometric indices were computed using WHO Anthro software, converting raw measurements (age, sex, and weight) into WAZ for children aged 6–59 months. Underweight was defined as WAZ < −2 SD based on WHO reference standards.

Chi‐square tests were used to assess baseline comparability of sociodemographic, environmental, childcare, and maternal health service‐related characteristics between transformed and nontransformed districts. Although differences in underweight prevalence were observed between the two groups, district transformation status was not independently associated with underweight in the multivariable analysis; therefore, pooled analysis was performed. For analysis, underweight was coded as a binary variable: 1 = underweight (WAZ < −2 SD) and 0 = not underweight (WAZ ≥ −2 SD).

Missing values were verified and corrected using the original questionnaires. Descriptive statistics were generated using frequencies, proportions, graphs, and cross‐tabulations. Bivariate analysis using chi‐square tests examined associations between independent variables and underweight. Prior to multivariable logistic regression analysis, multicollinearity among independent variables was assessed using variance inflation factors (VIFs). No evidence of problematic multicollinearity was observed, with all VIF values below 3.0. Variables with *p* < 0.25 in the bivariate analysis were entered simultaneously into the multivariable logistic regression model to control for potential confounders. Statistical significance was set at *p* < 0.05. Adjusted odds ratios (AORs) with 95% confidence intervals (CIs) were reported to indicate the strength of associations. Model fit was evaluated using the Hosmer–Lemeshow goodness‐of‐fit test (*p* = 0.33), indicating an acceptable fit.

### 2.11. Ethical Considerations

Ethical approval for the study was obtained from an institutional review board. Additional administrative permission was secured from the relevant local authorities prior to data collection. Participation was entirely voluntary, and written informed consent was obtained from all participants before enrollment. Participants received information in the local language regarding the study objectives, procedures, duration, and any potential risks and benefits. They were informed of their right to decline participation or withdraw from the study at any time without consequence. Confidentiality and privacy were ensured by excluding personal identifiers from all data collection tools and by securely storing and handling the study data.

## 3. Results

### 3.1. Sociodemographic Characteristics of the Study Participants

A total of 612 mother–child pairs participated, yielding a response rate of 97.6%. The nonresponse was primarily due to the absence of eligible participants during repeated household visits. About half were from transformed districts (309, 50.5%) and nearly half from nontransformed districts (303, 49.5%). The mean age of children was 30.6 ± 15.1 months in transformed districts and 27.1 ± 16.3 months in nontransformed districts. Male children comprised roughly half in both groups (49.8% in transformed vs. 51.5% in nontransformed districts). Maternal education was higher in transformed districts, with about half of mothers (49.0%) having no formal education compared with nearly three‐quarters (74.6%) in nontransformed districts. Enrollment in CBHI was also higher in transformed districts (84.0% vs. 58.0%). Mothers’ occupations varied: Most were farmers, especially in nontransformed districts (89.1% vs. 59.9%), while nearly one‐third of mothers in transformed districts were merchants (31.4% vs. 6.3%), and a smaller proportion were housewives (8.7% vs. 4.6%). Most mothers were married, about 85%–90% across districts. Regarding child age, about 41% in transformed districts and 56% in nontransformed districts were aged 6–24 months. Other variables, including family size, father’s education, monthly income, and place of residence, are summarized in Table 1.

**TABLE 1:** Socioeconomic and demographic characteristics of households in nontransformed and transformed districts, East Gojjam Zone, Ethiopia.

Variables	Categories	Nontransformed districts (*n* = 303) Frequency (%)	Transformed district (*n* = 309) Frequency (%)	Total (*n* = 612) Frequency (%)
Residence	Rural	285 (94.1%)	248 (80.2%)	533 (87.1%)
	Urban	18 (5.9%)	61 (19.7%)	79 (12.9%)

Age of the child (months)	6–24 months	169 (55.8%)	125 (40.5%)	294 (48.0%)
	25–59 months	134 (44.2%)	184 (59.5%)	318 (52.0%)

Sex of the child	Male	156 (51.5%)	154 (49.8%)	310 (50.7%)
	Female	147 (48.5%)	155 (50.0%)	302 (49.3%)

Marital status of mother	Married	274 (90.4%)	262 (84.8%)	536 (87.6%)
	Divorced	19 (6.3%)	33 (10.7%)	52 (8.5%)
	Widowed	10 (3.3%)	14 (4.5%)	24 (3.9%)

Mother’s occupation	Farmer	270 (89.1%)	185 (59.9%)	455 (74.3%)
	Merchant	19 (6.3%)	97 (31.4%)	116 (19%)
	Housewife	14 (4.6%)	27 (8.7%)	41 (6.7%)

Religion	Orthodox Christianity	294 (97.0%)	223 (72.2%)	517 (84.5%)
	Muslim	9 (3.0%)	86 (27.8%)	95 (15.5%)

Mother’s education	Have no formal education	226 (74.6%)	152 (49%)	378 (61.8%)
	Primary education	46 (15.2%)	77 (24.9%)	123 (20.1%)
	Secondary education	12 (4.0%)	39 (12.6%)	51 (8.3%)
	Above secondary education	19 (6.3%)	41 (13.3%)	60 (9.8%)

Father’s education (n1, n2, n3)	Have no formal education	201 (66.1%)	121 (39.2%)	322 (52.6%)
	Primary education	56 (18.5%)	73 (23.6%)	129 (21.1%)
	Secondary education	7 (2.3%)	25 (8.1%)	32 (5.2%)
	Above secondary education	10 (3.3%)	43 (13.9%)	53 (8.7%)

Family size	≤ 5	241 (79.5%)	240 (77.7%)	481 (78.6%)
	> 5	62 (20.5%)	69 (22.3%)	131 (21.4%)

Family monthly income	Low income	147 (48.5%)	142 (46.0%)	289 (47.2%)
	High income	156 (51.5%)	167 (54.0%)	323 (52.8%)

CBHI enrollment	Yes	176 (58.0%)	260 (84.0%)	436 (71.2%)
	No	127 (42.0%)	49 (16.0%)	176 (28.8%)

*Note:* n1 = 274 (nontransformed), n2 = 262 (transformed), n3 = 536 (total).

Abbreviation: CBHI = community‐based health insurance.

### 3.2. Environmental Characteristics

Household environmental factors differed substantially between nontransformed and transformed districts (Table 2). Latrine coverage was higher in transformed districts, 208 (67.3%), compared with nontransformed districts, 72 (23.8%). Similarly, households with improved latrines were more common in transformed districts, 156 (50.5%), than in nontransformed districts, 20 (6.6%). Safe disposal of under‐five children’s feces was reported in 191 (64.4%) households in transformed districts, compared to 72 (23.8%) in nontransformed districts. Solid waste disposal pits were available in 134 (43.4%) households in transformed districts and 50 (16.5%) in nontransformed districts. Liquid waste disposal was reported in 128 (41.4%) households in transformed districts, compared with 43 (14.2%) in nontransformed districts. The proportion of households using protected drinking water sources was higher in transformed districts, 222 (71.8%), than in nontransformed districts, 102 (33.7%). Overall, households in transformed districts exhibited better environmental sanitation and access to safe water.

**TABLE 2:** Environmental characteristics of households in nontransformed and transformed districts, East Gojjam Zone, Ethiopia.

Variables	Categories	Nontransformed districts (*n* = 303), *n* (%)	Transformed district (*n* = 309), *n* (%)	Total (*n* = 612), *n* (%)
Presence of household latrine	Yes	72 (23.8%)	208 (67.3%)	280 (45.8%)
	No	231 (76.2%)	101 (32.7%)	332 (54.2%)

Type of latrine	Unimproved	52 (17.2%)	52 (16.8%)	104 (17.0%)
	Improved	20 (6.6%)	156 (50.5%)	176 (28.8%)

Solid waste disposal	Yes	50 (16.5%)	134 (43.4%)	184 (30.1%)
	No	253 (83.5%)	175 (56.6%)	428 (69.9%)

Liquid waste disposal	Yes	43 (14.2%)	128 (41.4%)	171 (29.9%)
	No	260 (85.8%)	181 (58.6%)	441 (72.1%)

Place of under‐five child excreta disposal	In the latrine	72 (23.8%)	191 (64.4%)	263 (43.0%)
	Elsewhere	231 (76.2%)	118 (35.6%)	349 (57%)

Household source of drinking water	Protected	102 (33.7%)	222 (71.8%)	324 (52.9%)
	Not protected	201 (66.3%)	87 (28.2%)	288 (47.1%)

### 3.3. Child Feeding Practices and Household Food Security

EBF was reported by 84.8% of children in nontransformed districts and 88.3% in transformed districts. Despite breastfeeding, a substantial proportion of children discontinued breastfeeding before 24 months (71.0% in nontransformed and 66.0% in transformed districts). Dietary diversity was largely inadequate in both groups, with 74.9% of children in nontransformed districts and 72.5% in transformed districts consuming fewer than four food groups in the preceding 24 h. Participation in monthly growth monitoring and promotion (GMP) programs was higher in transformed districts (46.3%) compared to nontransformed districts (28.4%). Recent diarrhea was reported in 34.3% of children in nontransformed districts and 22.0% in transformed districts. Household food insecurity affected 56.8% of households in nontransformed districts and 51.1% in transformed districts. Other child care and feeding variables are summarized in Table 3.

**TABLE 3:** Child care and feeding practices of the respondents in nontransformed and transformed districts, East Gojjam Zone, Ethiopia.

Variables	Categories	Nontransformed districts (*n* = 303), *n* (%)	Transformed district (*n* = 309), *n* (%)	Total (*n* = 612), *n* (%)
Food distribution at home	Priority to child	173 (57.1%)	242 (78.3%)	415 (67.8%)
	Priority to others	130 (42.9%)	67 (21.7%)	197 (32.2%)

GMP monthly participation	Yes	86 (28.4%)	143 (46.3%)	229 (37.4%)
	No	217 (71.6%)	166 (53.7%)	383 (62.6%)

EBF duration	< 6 months	29 (9.6%)	20 (6.5%)	49 (8.0%)
	For 6 months	257 (84.8%)	273 (88.3%)	530 (86.6%)
	> 6 months	17 (5.6%)	16 (5.2%)	33 (5.4%)

EBF frequency	< 10 times	138 (45.5%)	87 (28.2%)	225 (36.8%)
	≥ 10 times	165 (54.5%)	222 (71.8%)	387 (63.2%)

Duration of breastfeeding	< 24 months	215 (71.0%)	204 (66.0%)	419 (68.5%)
	≥ 24 months	88 (29.0%)	105 (34.0%)	193 (31.5%)

Complementary food starts ^†^	< 6 months	22 (7.3%)	20 (6.5%)	42 (6.9%)
	At 6 months	237 (78.2%)	264 (85.4%)	501 (81.9%)
	> 6 months	17 (5.6%)	16 (5.2%)	33 (5.4%)

Frequency of food intake^∗∗^	< 3 meals per day	131 (43.2%)	105 (34.0%)	236 (38.6%)
	≥ 3 meals per day	145 (47.9%)	195 (63.1%)	340 (55.6%)

Dietary diversity score^∗^	Adequate	49 (16.2%)	76 (24.6%)	125 (20.4%)
	Inadequate	227 (74.9%)	224 (72.5%)	451 (73.7%)

Diarrhea	Yes	104 (34.3%)	68 (22.0%)	172 (28.1%)
	No	199 (65.7%)	241 (78.0%)	440 (71.9%)

HHFS status	Food secure	131 (43.2%)	151 (48.9%)	282 (46.1%)
	Food insecure	172 (56.8%)	158 (51.1%)	330 (53.9%)

Abbreviations: EBF, exclusive breastfeeding; GMP, growth monitoring and promotion; HHFS, household food security.

^†^Denominators for “Complementary food started” indicate children with valid responses: n1 = 276 (nontransformed), n2 = 300 (transformed), n3 = 576 (total).

^∗^Denominators for “Dietary diversity score” indicate children with valid responses: n1 = 276 (nontransformed), n2 = 300 (transformed).

^∗∗^Denominators for “Frequency of food intake” indicate children with valid responses: n1 = 276 (nontransformed), n2 = 300 (transformed), n3 = 576 (total).

### 3.4. Maternal and Child Health Characteristics

The mean age of mothers was 30.3 ± 5.88 years in nontransformed districts and 33.3 ± 5.82 years in transformed districts. ANC attendance was higher among mothers in transformed districts, where 298 (96.0%) reported at least one ANC visit, compared with 226 (74.5%) in nontransformed districts. Among mothers who attended ANC, nutrition counseling during pregnancy was received by 254 (82.2%) in transformed districts and 137 (45.2%) in nontransformed districts. Place of delivery differed substantially between the two groups. Facility‐based delivery was reported by 254 (82.2%) mothers in transformed districts, whereas only 138 (45.5%) mothers in nontransformed districts delivered in health facilities. PNC follow‐up was also more common in transformed districts, reported by 247 (79.9%) mothers, compared with 67 (22.1%) in nontransformed districts. Consumption of extra food during pregnancy was markedly higher among mothers in transformed districts, 229 (74.1%), than in nontransformed districts, 48 (15.8%). In both groups, most mothers had a birth order of one to four children, reported by 273 (90.1%) in nontransformed districts and 266 (86.1%) in transformed districts. Similarly, the majority of mothers reported a birth interval of 3 years or more, accounting for 86.5% in nontransformed districts and 88.0% in transformed districts.

Baseline comparisons using chi‐square tests showed significant differences between transformed and nontransformed districts across several sociodemographic, environmental, childcare, and maternal health service‐related characteristics, including residence, parental education, CBHI enrollment, sanitation conditions, source of drinking water, growth monitoring participation, consumption of additional food during pregnancy, ANC attendance, PNC follow‐up, and facility‐based delivery (all *p* < 0.05).

### 3.5. Prevalence of Childhood Underweight: Comparisons

**FIGURE 1:**
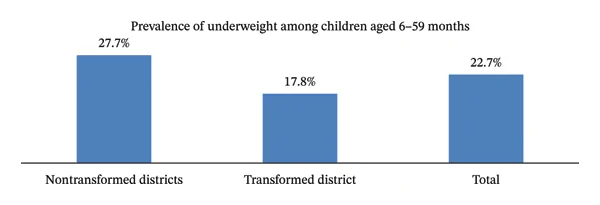
Prevalence of underweight among children aged 6–59 months by district status in East Gojjam Zone, Ethiopia.

The overall prevalence of underweight among children aged 6–59 months in the study area was 22.7% (*n* = 139; 95% CI: 19.0%–26.0%). When stratified by district status, the prevalence was higher in nontransformed districts at 27.7% (*n* = 84; 95% CI: 23.0%–33.0%) compared with 17.8% (*n* = 55; 95% CI: 14.0%–22.0%) in transformed districts (Figure 1).

In the unadjusted analysis, the prevalence of underweight differed significantly between nontransformed and transformed districts (*χ* ^2^ = 8.58, *p* = 0.003). However, in the multivariable analysis, district status was not independently associated with child underweight. Children residing in nontransformed districts had a comparable likelihood of being underweight to those in transformed districts (AOR = 1.06; 95% CI: 0.63–1.79). Because district status was not statistically significant after adjustment, subsequent analyses were conducted using pooled data.

### 3.6. Factors Associated With Childhood Underweight: Pooled Analysis

All independent variables were first assessed using bivariable logistic regression. Variables with a *p* value < 0.25, including district status, maternal education, CBHI membership, source of drinking water, intrahousehold food distribution, GMP participation, history of diarrhea, monthly household income, frequency of food intake, dietary diversity score, and household food security status, were simultaneously entered into the multivariable logistic regression model using the enter method.

After adjusting for potential confounders, five factors were significantly associated with underweight. Children from households using unprotected drinking‐water sources had higher odds of being underweight than those using protected sources (AOR = 1.82; 95% CI: 1.10–2.99). Similarly, children living in food‐insecure households were more likely to be underweight compared to those from food‐secure households (AOR = 2.67; 95% CI: 1.60–4.46). Recent diarrhea in the 2 weeks preceding data collection was also associated with higher odds of underweight (AOR = 2.49; 95% CI: 1.56–3.98). Furthermore, children from households that prioritized food distribution to other family members had increased odds of being underweight compared to those in households prioritizing children (AOR = 1.64; 95% CI: 1.02–2.65). Finally, children from low‐income households had higher odds of being underweight than those from high‐income households (AOR = 3.77; 95% CI: 2.34–6.08).

Variables such as district status, CBHI membership, dietary diversity, GMP attendance, frequency of food intake, and maternal education were not significantly associated with underweight in the adjusted model (Table 4).

**TABLE 4:** Factors associated with underweight among children aged 6–59 months in East Gojjam Zone, Ethiopia.

Variables	Overall category	*N*	Underweight (%)	COR (95% CI)	AOR (95% CI)
		612	22.7	—	—
District status	Nontransformed	84	27.7	1.77 (1.20–2.60)	1.06 (0.63–1.79)
	Transformed	55	17.8	Ref.	Ref.

Source of drinking water	Not protected	92	31.9	2.76 (1.86–4.11)	1.82^∗^ (1.10–2.99)
	Protected	47	14.5	Ref.	Ref.

CBHI membership	No	56	31.8	1.98 (1.33–2.95)	1.33 (0.80–2.20)
	Yes	83	19.0	Ref.	Ref.

DDS (*n* = 576)	Inadequate	117	25.8	2.56 (1.44–4.58)	1.83 (0.91–3.65)
	Adequate	15	12.0	Ref.	Ref.

Household food security status	Food insecure	110	33.3	4.36 (2.78–6.82)	2.67^∗∗^ (1.60–4.46)
	Food secure	29	10.3	Ref.	Ref.

GMP monthly participation	No	104	27.3	2.06 (1.35–3.15)	1.09 (0.63–1.87)
	Yes	35	15.2	Ref.	Ref.

Had diarrhea	Yes	68	39.5	3.39 (2.28–5.05)	2.49^∗∗^ (1.56–3.98)
	No	71	16.1	Ref.	Ref.

Frequency of food intake	< 3 times	69	29.4	1.85 (1.25–2.75)	1.14 (0.71–1.82)
	≥ 3 times	62	18.3	Ref.	Ref.

Food distribution	Priority to others	64	32.5	2.18 (1.47–3.21)	1.64^∗^ (1.02–2.65)
	Priority to child	75	18.1	Ref.	Ref.

Monthly income	Low	104	36.0	4.62 (3.02–7.07)	3.77^∗∗^ (2.34–6.08)
	High	35	10.8	Ref.	Ref.

Education status of mother	Have no formal education	91	24.1	3.48 (1.34–8.97)	1.46 (0.51–4.18)
	Primary education	30	24.4	3.54 (1.30–9.68)	2.04 (0.66–6.26)
	Secondary education	13	25.5	3.76 (1.23–11.4)	2.24 (0.65–7.67)
	Above secondary education	5	8.3	Ref.	Ref.

Abbreviations: ANC = antenatal care, CBHI = community‐based health insurance, DDS = dietary diversity score, GMP = growth monitoring and promotion, Ref. = reference.

^∗^ *p* < 0.05.

^∗∗^ *p* < 0.01.

## 4. Discussion

In this study, the overall prevalence of underweight among children aged 6–59 months was 22.7% (95% CI: 19.0%–26.0%). This estimate is comparable to findings from several Ethiopian settings, including Takusa District, Lalibela Town, the Tigray Region, Wonsho District, and the Ethiopian Mini EDHS 2019, where prevalence ranged from 19.5% to 25.6% [5, 11, 30–32]. The consistency across these diverse settings suggests shared underlying determinants, such as socioeconomic constraints, household food insecurity, and suboptimal child feeding practices. Although the unadjusted prevalence of underweight was higher in nontransformed districts, district transformation status was not independently associated with underweight after controlling for confounding factors, suggesting that household‐ and child‐level determinants play a more decisive role. This finding suggests that the apparent differences observed between transformed and nontransformed districts may be largely explained by underlying household, maternal, and child‐related characteristics rather than district transformation status itself. While the district transformation framework is intended to strengthen primary healthcare and community health services, improvements in child nutritional outcomes may depend on broader socioeconomic and household‐level conditions, including food security, caregiving practices, and access to safe water. Furthermore, because only one transformed district and two nontransformed districts were included in the study, unmeasured district‐level contextual factors may also have contributed to the observed differences.

Conversely, the prevalence observed here was higher than reports from Debretabor Town, Sodo Zuria District, and Gozamin District (14.5%–17.4%) [16, 17, 33] and lower than estimates from Medebay Zana District and the broader Amhara Region (26.7%–45.3%) [11, 34]. These disparities may be explained by several factors. Child nutritional status is highly sensitive to seasonal fluctuations, with higher undernutrition reported during lean seasons [35]. Household‐level variation, including maternal education, WASH access, and wealth, also influences the clustering of underweight. Furthermore, differences in public health service coverage and access to nutrition interventions across districts contribute to variation [36, 37]. Additionally, methodological heterogeneity in sampling, ecological zones, and assessment protocols can produce divergent estimates. These contextual variations underscore the necessity for localized studies to identify community‐specific drivers of undernutrition.

Multivariable analysis identified several factors significantly associated with underweight, spanning both environmental/health and socioeconomic domains. Children from households relying on unprotected water sources had higher odds of underweight compared to those using improved or protected water sources. One possible explanation is that unsafe or unimproved water increases exposure to waterborne pathogens, elevating the risk of diarrheal illnesses, which can impair nutrient absorption and lead to nutrient and fluid loss, thereby contributing to poor growth and underweight status. Evidence from multicountry analyses shows that children exposed to contaminated drinking water have a higher likelihood of being underweight compared to those with clean water sources, likely due to repeated enteric infections and compromised nutrient utilization [38]. Similarly, a recent episode of diarrheal illness significantly increased the odds of underweight in children. This association aligns with the well‐established bidirectional relationship between diarrhea and undernutrition, where undernutrition weakens immune defenses and increases susceptibility to infections, and diarrheal episodes further exacerbate nutrient loss and weight loss [39].

Regarding socioeconomic factors, household food insecurity was associated with underweight in children, consistent with evidence that limited access to sufficient and nutritious foods directly compromises a child’s growth and development. Food insecurity restricts both the quantity and quality of dietary intake, reducing energy and nutrient availability needed for normal weight gain and physical development. Studies in Ethiopia and other low‐ and middle‐income settings have found that children from food‐insecure households are significantly more likely to be underweight compared to those from food‐secure households, suggesting that inadequate food access is a key underlying determinant of undernutrition [40]. Low household income was also a significant determinant of underweight, reflecting how limited financial resources constrain a family’s ability to purchase diverse and nutrient‐rich foods. Poor economic status has been repeatedly linked with higher odds of underweight in children, as it affects food security, healthcare access, and caregiving practices, which are all critical components in preventing undernutrition [41]. Finally, children in households where food distribution prioritized other members over children had higher odds of underweight. This highlights the importance of intra‐household allocation patterns, where decisions about who eats first or who gets larger or higher‐quality portions can influence nutritional outcomes. Empirical evidence shows that in contexts of limited resources or food insecurity, food allocation often reflects social norms, bargaining power, and household roles, which may disadvantage certain members (including young children) and compromise their nutritional intake [42].

Together, these findings underscore the multifaceted nature of the socioeconomic determinants of underweight. The observed associations are consistent with evidence from Sub‐Saharan Africa demonstrating the central role of household food insecurity in child malnutrition [43] and are further supported by the UNICEF Conceptual Framework on Maternal and Child Nutrition, which identifies household food security, caregiving resources, and the household environment as key underlying determinants of child nutritional status [44]. Accordingly, the present study highlights the need for integrated, multisectoral interventions tailored to local contexts, focusing on improving household economic resilience, food security, equitable intrahousehold food allocation, access to safe drinking water, and the prevention and timely management of childhood illnesses. Strengthening nutrition‐sensitive programs within the health sector, expanding WASH interventions, and enhancing community‐based child health services may contribute to reducing underweight among children. Future longitudinal and intervention‐based studies are warranted to better understand causal pathways and evaluate the effectiveness of district transformation initiatives on child nutritional outcomes.

### 4.1. Strengths and Limitations

This study has several strengths, including its community‐based design, adequate sample size, and the use of standardized anthropometric measurements, which enhance the reliability of the estimated prevalence of underweight. In addition, the inclusion of key environmental, socioeconomic, dietary, and health‐related variables allowed for a comprehensive assessment of factors associated with underweight in the study area. However, some limitations should be considered when interpreting the findings. Information on caregiving practices and recent childhood illnesses relied on caregiver self‐report and may be subject to recall and social desirability bias despite careful data collection procedures. The cross‐sectional nature of the study precludes establishing temporal or causal relationships. In addition, only one transformed district and two nontransformed districts were included in the study. Although districts were selected randomly within each stratum, some observed differences may reflect unmeasured district‐specific contextual characteristics rather than transformation status alone, which may limit the generalizability of the findings. Furthermore, because only a small number of districts were included, adjustment for clustering at the district level was not feasible; therefore, some residual within‐cluster correlation may have remained. Although several important covariates were included in the analysis, residual confounding and measurement error cannot be completely excluded despite the use of standardized procedures and quality‐control measures. Finally, data were collected during a single period and may not fully capture seasonal variations in household food availability and child nutritional status.

## 5. Conclusions

The prevalence of underweight among children in the study area (22.7%) remains a significant public health concern. Based on WHO thresholds, this prevalence falls within the category of high public health significance. Several modifiable factors were found to be associated with being underweight, including the use of unprotected drinking water sources, household food insecurity, low household income, recent diarrheal illness, and intrahousehold food allocation practices that disadvantage children. Addressing underweight in this context requires a coordinated, multisectoral response that prioritizes improving access to safe water, strengthening household food security and economic resilience, promoting equitable child feeding practices, and enhancing the prevention and management of childhood illnesses, particularly diarrhea, within the primary healthcare system. These findings highlight the need for integrated nutrition‐ and health‐sensitive interventions to reduce childhood undernutrition in similar settings.

**Nomenclature**
ANC: Antenatal care
AOR: Adjusted odds ratio
CBHI: Community‐based health insurance
CI: Confidence interval
COR: Crude odds ratio
DDS: Dietary diversity score
EDHS: Ethiopian Demographic and Health Survey
EBF: Exclusive breastfeeding
HFIAS: Household Food Insecurity Access Scale
HHFS: Household food security
HSTP: Health Sector Transformation Plan
MOH: Ministry of Health
SD: Standard deviation
SPSS: Statistical Package for the Social Sciences
UNICEF: United Nations Children’s Fund
WAZ: Weight‐for‐age Z‐score
WHO: World Health Organization

## Author Contributions

Berhanu Abebaw Mekonnen and Kassahun Bishaw Anagaw conceived and designed the study, supervised data collection, and carried out data analysis and interpretation. Berhanu Abebaw Mekonnen took primary responsibility for drafting and revising the manuscript. Netsanet Fentahun assisted with data interpretation and critically revised the manuscript for important intellectual content.

## Funding

No funding was received to conduct the study.

## Disclosure

All authors read and approved the final manuscript for submission.

## Ethics Statement

Ethical approval for this study was obtained from the Institutional Review Board of the College of Medicine and Health Sciences, Bahir Dar University (protocol number: 476/2022).

## Consent

Written informed consent was obtained from all parents, caregivers, or legal guardians of the participating children after providing a clear explanation of the study objectives, procedures, and potential risks and benefits. All study procedures were conducted in accordance with national ethical guidelines and the principles of the Declaration of Helsinki.

## Conflicts of Interest

The authors declare no conflicts of interest.

## Data Availability

The datasets generated and analyzed during the current study are available from the corresponding author upon reasonable request.
